# Chest Pain as a presenting complaint in patients with acute myocardial infarction (AMI)

**DOI:** 10.12669/pjms.292.2921

**Published:** 2013-04

**Authors:** Muhammad Ajmal Malik, Shahzad Alam Khan, Sohail Safdar, Ijaz-Ul-Haque Taseer

**Affiliations:** 1 Dr. Muhammad Ajmal Malik, MBBS, MD, Senior Registrar, Nishtar Hospital Multan, Pakistan.; 2 Dr. Shahzad Alam Khan, FCPS, Senior Registrar, Nishtar Hospital Multan, Pakistan.; 3 Mr. Sohail Safdar, M. Sc, Research Officer, PMRC Research Centre, Nishtar Medical College, Multan, Pakistan.; 4 Dr. Ijaz-Ul-Haque Taseer, MBBS, MD, Chief Research Officer, PMRC Research Centre, Nishtar Medical College, Multan, Pakistan.

**Keywords:** Chest pain, acute myocardial infarction, Precordial chest pain

## Abstract

***Objective:*** To study various characteristics of chest pain in acute myocardial infarction patients.

***Methodology:*** A total of 331 patients of AMI admitted at Cardiology unit Nishtar Hospital Multan and Chaudhry Pervez Elahi Institute of Cardiology Multan, irrespective of the age and gender, were included in this study. The study duration was one year starting from June 2011 to June 2012. Non-probability purposive sampling technique was used in this descriptive study. Informed consent to participate in this study was taken. Data were entered and analyzed using SPSS-11.

***Results:*** A total number of 331 patients with AMI were included in the study. Mean age was 54.99±11.25 years with minimum age 20 years and maximum age 90 years. It included 264(79.8%) male and 67(20.2%) female patients with male to female ratio of 3.9:1. Out of these 331 patients 308 (93.1%) patients reported chest pain as the presenting complaint. Remaining 23(6.9%) presented with clinical features other than chest pain. There were 127(38.4%) patients with pre-cordial chest pain, 115(34.7%) had retrosternal chest pain, 58(17.5%) were having epigastric pain. Severe chest pain was seen in 281(84.9%) patients while 26(7.9%) had only mild chest discomfort. Radiation of the pain to shoulder, neck and jaw was seen in 75 (22.7%) patients. In 42(12.7%) patients, pain radiated to both sides of chest. Another 55(16.6%) patients had pain radiation to chest, shoulder, upper arm and ulnar side of left forearm. Chest pain radiation to interscapular region along with both sides of chest was present in 10(3.0%) patients. In 11(3.3%) patients’ pain radiated only to left side of chest. Pain persisting for >20 minutes was reported by 298 (90%) patients while only 10(3.1%) had pain persisting for <20 minutes.

***Conclusion:*** There is considerable overlap in chest pain of cardiac as well as non cardiac causes. However, vigilant evaluation of characteristics of chest pain in history taking may help to overcome this dilemma. Severe and prolonged precordial chest pain in a male patient between the age of 41-70 years, with pain radiation to left shoulder, neck and jaw is highly suggestive of AMI.

## INTRODUCTION

Acute myocardial infarction (AMI) is a cardiac emergency. The clinical diagnosis of AMI requires an integrated assessment of the history especially with reference to chest pain along with some combination of indirect evidences of myocardial infarction using biochemical, electrocardiographic, and imaging modalities. In the United States, nearly one million patients suffer from AMI per year.^1^ Even in Pakistan 46 % of the deaths are due to myocardial infarction and 27% are due to other subsets of Ischemic heart disease.^2^

Chest pain is the most common presenting complaint of acute myocardial infarction. The classic manifestation of ischemia is usually described as a heavy chest pressure or squeezing, a “burning” feeling, or difficulty in breathing. The discomfort or pain often radiates to the left shoulder, neck, or arm. Chest pain may be atypical in few cases. It builds in intensity over a period of few minutes. The pain may begin with exercise or psychological stress, but acute myocardial infarction most commonly occurs without obvious precipitating events.

Each year five million patients come to emergency departments with chest pain.^3^ However, diagnostic evaluation reveals that only 15 to 25 percent of patients with acute chest pain actually have acute coronary syndrome.^4^^,^^5^ The difficulty is to discriminate patients with acute coronary syndrome from those with non-cardiac chest pain. Pope et al found that only 2.1 percent of patients with chest pain having acute myocardial infarction were discharged from the emergency department.^6^ Patients with acute myocardial infarction who are mistakenly discharged from the emergency department have short-term mortality rates of about 25 percent, at least twice what would be expected if they were admitted.^7^

It is therefore of utmost importance to emphasize the evaluation of chest pain and to discriminate chest pain of acute myocardial infarction from non-cardiac chest pain. By doing this, we can eliminate the chances of mistaken discharge of patients with acute myocardial infarction having initial normal ECG. We can also decrease undue burden on health personnel by avoiding mistaken admission of those patients who do not actually have myocardial infarction or acute coronary syndrome.

So the present study was conducted to find out the characteristic and peculiar features of chest pain which can ultimately help in diagnosis of AMI.

## METHODOLOGY

A total of 331 patients of AMI admitted at Cardiology unit Nishtar Hospital Multan and Chaudhry Pervez Elahi Institute of Cardiology Multan, irrespective of the age and gender, were included in this study. The study duration was one year starting from June 2011 to June 2012. Non-probability purposive sampling technique was used in this descriptive study. Informed consent to participate in this study was taken. A pre-designed questionnaire was used to record the data. Data were entered and analyzed using SPSS-11.

## RESULTS

A total number of 331 patients with AMI were included in the study. Mean age was 54.99±11.25 years with minimum age 20 years and maximum age 90 years. It included 264(79.8%) male and 67(20.2%) female patients with male to female ratio of 3.9:1. Out of these 331 patients 308 (93.1%) patients reported chest pain as the presenting complaint. Remaining 23(6.9%) presented with clinical features other than chest pain.

Majority of the patients i.e. 278(83.98%) were between the age of 41-70 years. There were 22 (6.64%) patients between the age of 71-90 years. Only 5(1.51%) were 30 years and below. (Table-I)

**Table-I:** Age wise distribution of AMI (n = 331

*Age Group*	*Frequency*	*Percentage*
20-30	5	1.51
31-40	26	7.85
41-50	106	32.03
51-60	107	32.33
61-70	65	19.64
71-90	22	6.64
*Total*	*331*	*100*

There were 127(38.4%) patients with pre-cordial chest pain, 115(34.7%) had retrosternal chest pain, 58(17.5%) were having epigastric pain and only 2(0.6%) had pain in the back of chest as initial symptom. Only 3 (0.9%) patients had pain both in epigastrium and retrosternum. Severe chest pain was seen in 281(84.9%) patients while 26(7.9%) had only mild chest discomfort.

Radiation of the pain to shoulder, neck and jaw was seen in 75 (22.7%) patients. In 42(12.7%) patients pain radiated to both sides of chest. Another 55(16.6%) patients had pain radiation to chest, shoulder, upper arm and ulnar side of left forearm. Chest pain radiation to interscapular region along with both sides of chest was present in 10(3.0%) patients. In 11(3.3%) patients’ pain radiated only to left side of chest. Another 11(3.3%) had pain radiation to ulnar side of arm only. Radiation of pain to the left shoulder alone was present in 16(4.8%), to interscapular region alone in 9 (2.7%) and to jaw alone in 4(1.2%) patients and 68 (20.5%) did not describe radiation to any site.

Pain persisting for >20 minutes was reported by 298 (90%) patients while only 10(3.1%) had pain persisting for <20 minutes. Ninety two (27.8%) patients had sensation of heavy weight over chest. Pain was constricting in 36 (10.6%), choking in 30(9.6%), burning in 48 (14.5%) and stab like in 42 (12.7%) patients. Only 2(0.6%) had reported both choking and constricting pain while, another 2(0.6%) had choking as well as burning character chest pain. Acute myocardial infarction occurred in morning time in 128(38.7%) patients, 98 (29.6%) patients had AMI in evening while in 96(29.0 %) AMI occurred at night and 9 (2.7%) patients could not clearly describe the timing of onset.

## DISCUSSION

Despite of all advances in the management of cardiovascular diseases, yet discrimination between chest pain due to AMI and non- cardiac chest pain remains a dilemma. Unfortunately, not much literature is available about the characteristics of chest pain to differentiate these two conditions. Only three of the selected studies combined different signs and symptoms for the diagnosis of AMI.^8^^-^^10^

Age is an important determinant of AMI in patients having chest pain. Incidence of AMI increases with increasing age. In females age of presentation is even higher by 5-10 years.^11^ In our study, we found that majority of the patients were between the age of 41-70 years, while a study conducted by Malik et al, 85% of the patients were between 41-60 years of the age.^2^ Age of presentation was slightly higher in our population as compared to that which was noticed by British Heart Foundation i.e. 30-69 years.^12^ In Belgium Bartholomeeussen et al^13^ found that incidence of AMI is high at the ages between 45–75 years. Our results are in accordance with the study conducted by Bartholomeeussen et al.^13^ However the mean age for first MI among south Asian is lower compared to the individuals in other countries.^14^

At any given age, prevalence of coronary heart disease is greater in men than in women.^15^ Risk factors like hypertension and hyperlipidemia are more prominent for men than women in the late 40- to early 50-year range; then their prevalence is higher in women. Women have an extra protection during their early reproductive life due to the effect of sex hormones. In our study majority of the patients with AMI were male (79.8%). Studies conducted by Hafeez et al and Shabbir et al also showed male dominance.^16^^,^^17^ Albarran et al^18^ had also discovered that AMI is more common in males (68%) as compared to females (32%). Chirsten et al^19^ found AMI prevalence was 62% in males. In a local study conducted by Mujtaba et al^20^ at Karachi had also similar findings.

Site of the chest pain gives important clue to the diagnosis of ACS/AMI. Pain which is located in the center of chest is more likely to be ischemic than a peripherally located chest pain. We found that precordial chest pain is the most common site for chest pain. There were 127(38.4%) patients with precordial chest pain in our setting. De Silva also noticed that precordial and retrosternal sites are most common sites for chest pain in CAD.^21^ Bosner et al^22^ analysed 1212 patients (534 men and 678 women) for the aetiology of their chest pain; of those 180 patients (92 men and 88 women) were diagnosed as having CHD. Pain was present on the left side of chest in 56 (63.6%) females and in 63 (68.5%) males. Bosner et al^22^ noticed that chest pain was localized on the right side of the chest in 34.1% patients. However, in our settings none of the patient presented with right sided chest pain.

Most common site where AMI pain radiates is left shoulder and arm.^23^^,^^24^ This is because of presence of heart on the left of chest, so pain radiates along left sided cervical nerve roots. In our study 55(16.6%) patients had pain radiation to left shoulder, left upper arm and ulnar side of left forearm. Solt et al^25^ claimed a high prevalence of chest pain radiation to the jaw especially in females. However, we have noticed that only 4(1.2%) patients had pain radiation to the jaw alone but pain radiation to the jaw was present in combination with radiation to shoulder and neck in 22.7% patients.

Duration of chest pain more than 20 minutes can be taken as cutoff for AMI. In our study, it was found that 90% of patients had chest pain persisting for >20 minutes. Similar results have been proven in multiple other international studies.^26^^,^^27^ However those attacks of chest pain that are not very severe or prolonged, but distressing enough for patients to contact a general practitioner, present a more difficult problem in diagnosis and management.^28^

Although chest pain is the most important symptom of AMI but it may be invariably absent in some patients. In our setting 6.9% patients had symptoms other than chest pain (painless AMI). In a study conducted by Hafeez et al pain less MI was seen in 6 % of the patients.^16^ Abidov et al^29^ also found that some patients may present with symptoms other than chest discomfort; such as “angina equivalent” symptoms include dyspnea (most common), nausea and vomiting, diaphoresis, and unexplained fatigue. Chest pain remains most important symptom of AMI but in few patients it may not be there. Further studies on large scale are required about the characteristics of chest pain favoring AMI.

## CONCLUSION

There is considerable overlap in chest pain of cardiac as well as non cardiac causes. However, vigilant evaluation of parameters of chest pain in history taking may help to overcome this dilemma. Severe and prolonged precordial chest pain in a male patient between the age of 41-70 years, with pain radiation to left shoulder, neck and jaw is highly suggestive of AMI.
